# Spontaneous Carotid Artery Dissection Presenting as Trigeminal Neuralgia in the Emergency Department

**DOI:** 10.5811/cpcem.2020.1.44664

**Published:** 2020-04-14

**Authors:** Robert Look, Thomas J. Terlau, Ryan Misek

**Affiliations:** * Midwestern University, Chicago College of Osteopathic Medicine, Department of Emergency Medicine, Downers Grove, Illinois; † Midwestern University, Chicago College of Osteopathic Medicine, Downers Grove, Illinois; ‡ Midwestern University, Chicago College of Osteopathic Medicine, Department of Clinical Education, Downers Grove, Illinois

**Keywords:** Carotid Artery Dissection, Trigeminal Neuralgia

## Abstract

**Introduction:**

Carotid artery dissection (CAD) is a critical diagnosis in the emergency department (ED). Trigeminal neuralgia, while not uncommon, may cause the patient significant discomfort but generally is not attributed to severe morbidity and mortality.

**Case Report:**

We present a case of spontaneous CAD presenting with the classic intermittent “lightning-like” jaw and head pain suggestive of trigeminal neuralgia that was ultimately diagnosed utilizing computed tomography angiogram after multiple visits to the ED.

**Discussion:**

Coincidentally the patient had been started on anticoagulation a few days prior and no additional intervention was required.

**Conclusion:**

This case report discusses current recommendations for diagnosis, treatment, and long-term prognosis of CAD.

## INTRODUCTION

Carotid artery dissection (CAD) occurs when the integrity of the arterial wall is compromised, allowing blood to collect between layers of the arterial wall forming an intramural hematoma. This can result in cerebral ischemia due to mechanical obstruction of blood flow or thromboembolism formation. CADs are categorized as spontaneous or traumatic based on history of antecedent trauma. Arterial dissections are a common cause of stroke in young patients; however, they can occur in all ages. We describe a patient who presented to the emergency department (ED) on two consecutive days with complaint of intermittent, sudden, “lightning-like,” left lower face pain. Initial non-contrast neuroimaging was unremarkable, and the patient did not have any objective neurological deficits. Upon second visit to the ED, her pain returned and was significantly worse. Computed tomography angiography (CTA) of head and neck revealed the patient had a left internal CAD. This case describes an atypical presentation of a potentially serious condition in a patient presenting with trigeminal neuralgia-type symptoms, which could have led to life-threatening consequences if not identified and treated.

## CASE REPORT

A 76-year-old Caucasian female with a past medical history of atrial fibrillation (not on anticoagulation), hypertension, chronic obstructive pulmonary disease, and obstructive sleep apnea presented to the ED complaining of intermittent, left-sided jaw and lower face pain onset that particular morning. She described her pain as “sharp and lightning-like,” causing her to wake up in the middle of the night. Patient reported similar symptoms three months prior after she had “dental work” completed; however, she denied any recent dental procedures. She also denied any other symptoms during initial presentation. Neurologic exam was normal, including cranial nerve testing, upper and lower extremity sensorimotor function, and cerebellar testing. The patient had mild tenderness over the left side of her mandible and the jaw opened and closed without palpable clicking or grinding. Vital signs revealed a temperature of 36.4° Celsius, heart rate of 105 beats per minute, respirations of 18 breaths per minute, blood pressure of 151/101 millimeters of mercury, and pulse oximetry of 96% on room air.

The differential diagnosis included acute myocardial infarction, trigeminal neuralgia, temporal arteritis, temporomandibular joint dysfunction, herpes zoster, subarachnoid hemorrhage, dentalgia, and other causes of acute jaw pain in an elderly female patient. CTA of the head and facial bones without intravenous (IV) contrast were grossly normal. Electrocardiogram revealed rate-controlled atrial fibrillation without acute ischemic changes. Chest radiograph was clear. Laboratory analysis revealed an erythrocyte sedimentation rate of 34 millimeters per hour (mm/hr) (reference range 0–30 mm/hr); the remainder of the complete blood count, complete metabolic panel, thyroid stimulating hormone, troponin-I, urinalysis, and magnesium level were unremarkable.

**CPC-EM Capsule**

What do we already know about this clinical entity?

Carotid artery dissection can present in a variety of ways, ranging from unilateral headache to Horner’s syndrome, and can be a severely debilitating disease.

What makes this presentation of disease reportable?

The pain associated with carotid artery dissection is usually not described as “lightning-like” jaw pain, mimicking trigeminal neuralgia or dental pain.

What is the major learning point?

Always expand your workup and differential diagnosis on patients who return multiple times for the same complaint, or with progression of disease process

How might this improve emergency medicine practice?

Including carotid artery dissection in our differential for unilateral jaw pain in patients with no intraoral, dental, or facial abnormalities on physical exam.

The patient was restarted on apixaban after speaking with her cardiologist, given her history of atrial fibrillation. Neurology was consulted about the possibility of these symptoms being representative of trigeminal neuralgia and starting carbamazepine; however, neurology requested no medication changes and for the patient to follow up with her dentist prior to outpatient neurology follow-up to rule out dental causes. Patient was ultimately discharged home with outpatient neurology and dental follow-up.

**Image 1:**
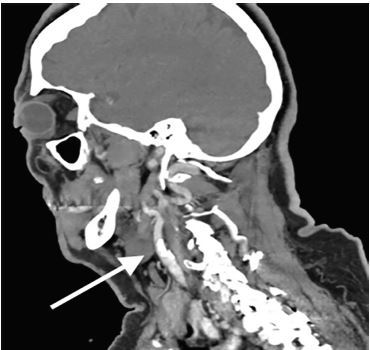
Sagittal view of computed tomography with angiography of the head and neck identifying an intimal flap in the left internal carotid artery consistent with a carotid artery dissection.

**Image 2:**
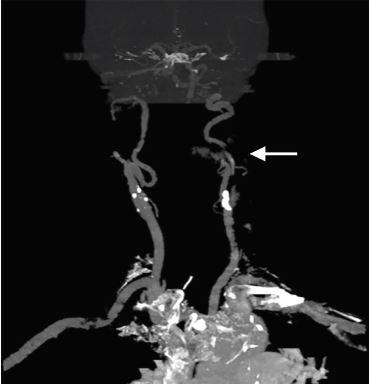
Coronal view of computed tomography with angiography of the head and neck identifying an irregular 50–60% narrowing of proximal mid-cervical segment of left internal carotid artery with intimal flap consistent with carotid artery dissection.

The patient returned to the ED with complaints of similar pain two days later; however, this time her left lower face and jaw pain was present leaving the patient in moderate to severe distress. Given the repeat visit and failure of outpatient therapy, early neurology consultation was obtained regarding analgesic recommendations while the workup ensued. The consulting neurologist recommended methylprednisolone 250 milligrams (mg) IV and ketorolac for analgesia. The patient’s pain remained refractory and IV fentanyl was given; we considered other diagnoses including CAD. CTA of the head and neck was obtained, which revealed a CAD of the left internal carotid artery (Images 1 and 2). The patient still did not exhibit any neurologic findings. Neurosurgeons whom we consulted at a tertiary-care hospital recommended continuing anticoagulation with close follow-up at their outpatient specialty clinic.

During hospitalization, the patient did not develop any neurologic deficits. Pain was controlled with IV hydromorphone and methylprednisolone 125 mg every 12 hours. She was ultimately discharged within 24 hours and continued on oral apixaban and steroids.

## DISCUSSION

This patient’s presenting symptoms resembled trigeminal neuralgia. Had the CTA not been performed, the CAD might have been missed. It is unclear whether the CAD was the true etiology of the patient’s “lightning-like” pain or was merely a concomitant or incidental finding.

The average annual incidence rate for spontaneous internal CAD is 1.72 per 100,000 population (95% confidence interval, 1.13 to 2.32).^1^ However, incidence is thought to be underestimated due to many patients being asymptomatic.^1^ While the pathophysiology of CAD is not well understood, a common theory is a multifactorial cause involving both genetic predisposition and environmental factors, such as infection or minor trauma acting as a trigger. Some evidence suggests that up to 50% of patients with CAD have autosomal dominant, skin connective-tissue abnormalities. 2 It is also common for patients with CAD to have concomitant arterial abnormalities suggesting a constitutional arteriopathy.^2^ The inciting trauma for CAD has been reported to be as minor as practicing yoga, painting a ceiling, coughing, sneezing, or vomiting.^3^ Many patients do not recall a trauma or inciting event.^3^ History of recent upper respiratory infection has been suggested as a potential trigger for dissections due to the seasonal variation of their incidence, peaking in the fall.^3^

The most frequently encountered symptoms in CAD are headache, neck pain, cerebral ischemia (transient ischemic attack or infarct), Horner syndrome, and cranial nerve palsies.^3^ Recent-onset unilateral headaches with continuous pain have been considered the most important paucisymptomatic of cervical (vertebral and carotid) artery dissections.^4^ The diagnostic criteria for headache attributed to cervical artery dissection includes two of the following: temporal pain that evolves to encompass other signs of cervical artery dissection; pain that improves/resolves after one month of onset or worsens contemporarily with other signs of cervical arterial lesions; pain that is severe and continues for days (most sensitive finding) or precedes signs of acute retinal/cerebral ischemia; and/or pain that is unilateral and ipsilateral to dissected cervical artery.^4^ Unilateral neck pain is seen in about 25% of patients, and unilateral facial/orbital pain in about 50% of patients.^3^ Approximately 10% of pain is isolated to the face or neck; most pain will progress to a unilateral headache in the temporal and frontal regions.^3^

Cerebral or retinal ischemic symptoms are reported in 50–95% of patients with a spontaneous dissection of the carotid artery and are commonly preceded by transient monocular blindness.^3^ Sudden onset Horner syndrome is seen in less than half of patients with CAD, but is considered to be specific for CAD when it occurs in conjunction with unilateral headache or facial pain. Cranial nerve palsies are a relatively rare finding, occurring in 7–12% of CAD cases.^2^,^3^ When they do occur, the hypoglossal nerve is most commonly affected, but the oculomotor, trigeminal, and facial nerves may also be involved. In a retrospective study of 245 patients who were diagnosed with spontaneous cervical artery dissections, only 8% presented with pain as their only symptom.^5^ The pain was most often described as having a gradual onset and progressing into a steady ache or sharp pain.^3^,^5^ However, four patients reported a sudden “thunderclap” headache at the presumed time of onset.^3^,^5^ Literature search by the authors did not identify any previously published reports of vertebral or CAD presenting with only the paroxysmal “lightning-like” pain resembling that of trigeminal neuralgia, making our patient’s presentation unique.

Diagnosis of CAD can be made on magnetic resonance imaging (MRI) with angiography, CTA, or conventional angiography, the latter of which has been largely replaced.^2^ MRI with angiography is generally the preferred method; however, CTA can be used when there is limited access to MRI, such as in the ED. The acute phase of CAD is treated with either anticoagulant or antiplatelet drug therapy to prevent ischemic events. Research indicating which is better is unclear and ongoing. 6 Treatment plans are largely made on a case by case basis.^6^ Prognosis is generally good with a mortality rate of less than 5%, and ischemic event recurrence rate of less than 13%.^2^

## CONCLUSION

Carotid artery dissection, while uncommon, is a critical diagnosis that emergency providers should consider in patients presenting with head, neck, or jaw pain with a focal neurological deficit. When no deficit is identified, it should still be considered when risk factors such as reported trauma and genetic factors are present. It is imperative that providers maintain a broad differential and consider CTA when this diagnosis is considered, especially in the patient with multiple presentations when a causative lesion contributing to their symptoms has not yet been identified.
